# Facts and Observations Relative to the External Application of Opium by Friction, so as to Be Absorbed by the Lymphatics

**Published:** 1799-07

**Authors:** M. Ward

**Affiliations:** Surgeon to the Manchester Infirmary


### Facts and Observations relative to the external Application of Opium by Friction, so as to be absorbed by the Lymphatics. By M. Ward, Surgeon to the Manchester Infirmary.

It appears to be not only a professional but a moral duty, to lay before the public, at an early period, improvements in the healing art, which are sufficiently well authenticated, and may be adopted by others, without impropriety or danger in similar cases. On this ground, I trust, the communication of the following facts and observations will be justified: and I am happy in being able to avail myself of the high authority of Mr. Pott; who, in his admirable tract on the mortification of the toes and feet, has thus sanctioned a novel mode of exhibiting opium. "If this was an experiment, in which the life, or limb, or health of the patient was in any degree endangered, or by which the person on whom it may be tried, could in any degree be injured, I should have withheld what I now publish, until a greater length of time and more experience had rendered it still more absolutely certain; and I should have thought myself vindicable in so doing: but, as this is a medicine whose general effects are well known, and which is at the same time, so capable of direction and management, that it is 1most impossible for any person who deserves to be trusted with medicine at all, to do any material harm with it, I thought it would be wrong and unjust to conceal what had occured to me, lest I might thereby deprive the afflicted of an assistance which, I verily believe, is not to be obtained from any other quarter." *

May 14, 1799, Mr. G. P. fourteen years of age, became delirious on or about the eleventh day from the commencement of typhus. Musk, opium, &c. were prescribed, both by myself, and afterwards by the advice of Dr. Percival, without his becoming composed or rational; except at intervals. On the contrary, the delirium increased till the seventeenth or eighteenth day, when he became extremely restless and turbulent, requiring two persons to keep him in bed. He continued in this state, till the evening of the twentieth or twenty-first day, when he seemed nearly exhausted. His feet were cold, his pulse weak and irregular, and he discharged his urine and stools involuntarily.

He had taken 5½ grains of opium, 54 of musk, and the same of volatile salt in the last 48 hours: but could not now retain them on his stomach.

Not having an opportunity of consulting with Dr. Percival at that time, and not supposing it possible for our patient to live more than an hour or two, unless

* Earle's edition of Pott's Works, Vol 3, page 364.

unlefs a fpeedy change could be effected; I directed fix grains of opium, very finely powdered, to be mixed with an ounce of lard, and to be divided into two equal parts: one of them to be rubbed on the infide of one leg, and the top of the foot, directly: and the other on the other leg and foot, in two or three hours afterwards. This practice was fuggefted to me, by an extract of a letter I had juft met with, in Duncan's Annals of Medicine for 1798, from Vin. Chiarugi, Phyfician to the hofpital of St. Boniface, at Florence, to Dr. L. Frank, on the effects of opium applied externally, in maniacal delirium, &c. This method of applying opium, was firft propofed, I believe, by Dr. Chiarenti, of Florence.

It occured to me, that as it had produced calmnefs and fleep in the inftances mentioned by Dr. Chiarugi, it might probably have the fame effect in a delirium arifing from fever: and feeing our patient in fo hopelefs a ftate, I was the more anxious to try it on that account.

May 15. At ten A. M. I was furprifed to find the patient better, and in a quiet comfortable fleep: and was informed that the attendants expected every gafp would have been his laft, till four o'clock, (fix hours after the firft portion of ointment was rubbed in) when he became a little calmer, and grew gradually more fo till eight; he then began to dofe, and at nine fell into an eafy fleep. Dr. P. and I vifited him at twelve, and were pleafed and furprifed to find him ftill afleep.

At ten P. M. he was perfectly compofed, and had flept from the time I laft faw him till half paft four, when he awoke, faid he was hungry, and afked for fome bread and butter, and tea: he then expreffed a wifh to have his bed made, fat up ten minutes, and eat an orange: and when removed to bed, fell afleep directly, and flept till eight; when he feemed uneafy, and had a flight return of the delirium. Six grains of opium were now rubbed in on one thigh, agreeably to the directions we had given. When the friction had been continued half an hour, he defired the nurfe to defift, and fell afleep immediately. His pulfe, which had hitherto varied from 100 to 120, was now only 80, but ftronger.

May 16. At 5 A. M. Mr. P. came to tell me, his fon had flept well till two; but was now reftlefs and uneafy, and that he had ufed the laft portion of the ointment.

Eight grains of opium, mixed with three drachms of lard, were directed to be rubbed in immediately.

Ten A. M. I was informed, he could not bear the friction longer than eight or ten minutes. He was now reftlefs; but not delirious; pulfe fcarcely

ſcarcely perceptible. Wine was preſcribed. At twelve, Dr. P. found him in the ſame ſtate, and preſcribed tinct. opii. gr. xv. 4ta quaq. horâ. At ten, P. M. he was eaſy and rational; but had not ſlept much; pulſe 92; tongue much cleaner; ſkin cool. Nine grains of opium, mixed with three drachms of lard, were now ordered to be applied, and the ſame quantity to be repeated in four hours, unleſs he became compoſed.

May 17, twelve o'clock. The firſt portion of ointment only was applied; and he ſoon grew tired of the friction, ſo that very little could have been abſorbed. I was aware of this being likely to happen, and therefore had ordered an increaſed quantity of opium. He was now calm and rational; but had not ſlept well. Pulſe 100. Omit. ung.—Contin. tinct. opii. At eight, P. M. pulſe 96; quite eaſy. Contin. tinct. opii.

May 18. Slept well laſt night; appetite improved; tongue clean; pulſe 96, regular, and ſomewhat ſtronger.

May 20th. Sleeps well, and improves rapidly.

The quantity of opium applied externally, was 35 grains, and this was rubbed on the legs and thighs, in the courſe of the lymphatics, at ſix different times, in forty-eight hours; not in equal proportions, or at regular intervals; but whenever he began to grow uneaſy.

The opium was powdered exceedingly fine.

This hiſtory I communicated to Dr. Percival; from whom I have ſince received the following caſes and remarks; which I ſhall deliver in his own words.

" The narrative you read to me this morning, I believe to be perfectly accurate; and I now ſend, agreeably to your requeſt, a brief ſtatement of the two caſes I mentioned to you, in which opium was externally applied with much apparent benefit.

" You have a juſt claim to this communication; becauſe it was from my being in ſome degree perſuaded of the ſucceſs of the practice you purſued in the inſtance of our patient, Mr. P jun. that I was induced to adopt the ſame mode of treatment.

" May 16, 1799. I was called to viſit Mrs. ———, a lady about forty-ſix years of age, ſubject to epileptic fits, to anomalous gout, and to temporary mental derangement. I found her in a ſtate of high delirium, without fever. Having remarked that, under ſimilar circumſtances, the internal exhibition of laudanum aggravated the affection of her head, probably by producing inebriation, I regarded this as a peculiarly favorable opporopportunity of trying the uſe of opium externally. I therefore directed three drachms of laudanum to be mixed with an equal quantity of olive oil, by the yolk of an egg, and to be diligently rubbed into the legs. The friction was ſoothing and grateful to her; and in a few hours, ſhe was obſerved to become conſiderably calmer. The like inunction was repeated at night.

" May 17. I found her perfectly compoſed and rational. She had enjoyed much refreſhing ſleep. Her pulſe was regular, her heat natural, and a gentle perſpiration had taken place ſoon after the firſt inunction.

" The ſame plan was continued.

" May 18. She remained perfectly compoſed and rational. During the ſpace of forty-eight hours, the inunction had been four times renewed. It was performed with due diligence and care; and the hand of the ſervant who rubbed in the liniment was covered with a ſmooth bladder.

" What proportion of the twelve drachms of laudanum, thus conſumed, may be ſuppoſed to have been conveyed by abſorption into the ſyſtem?

" May 17, 1799. Mr. ———, a gentleman between thirty and forty years of age, of a delicate make, had been confined by a low fever more than a week. Laſt night a delirium occurred, which progreſſively increaſed in violence; ſo that this morning I found him in a ſtate of maniacal fury, yet with a languid pulſe.

" A bolus of muſk, volatile ſalt and opium, was preſcribed. But the patient loudly and peremptorily refuſed not only medicine, but all ſuſtenance. It became neceſſary to confine him by a ſtrait waiſtcoat; and I directed his feet and legs to be well fomented. Three drachms of laudanum, mixed with a like proportion of olive-oil, were afterwards rubbed into them. Within the ſpace of five or ſix hours he grew more calm; and was prevailed upon to take nearly two glaſſes of wine.

" A repetition of the inunction was directed.

" May 18. The patient was in a ſtate of tolerable compoſure. He had taken both wine and nutriment. The inunction had been thrice renewed; but, as it had not produced much ſleep, I directed fifteen drops of laudanum to be adminiſtered every four or five hours, in coffee, of which he was ſaid to be fond, and which conceals the taſte of opium better than any other vehicle. The inunction was diſcontinued.

" May 19. The patient had enjoyed ſound and refreſhing ſleep; and awoke in the morning almoſt perfectly rational, with a conſiderable abatement of every ſymptom of fever.

*Additions*

*Additions to Dr.* Percival's *Communication.*

"In the introduction of new modes of treatment, it is incumbent on the medical practitioner, to be sedulously cautious, not only that he founds his trials on just analogies, but that he conducts them with impartiality, and records, with faithfulness, their good or ill success.

"The apparently happy effects of opium in the case of Mr. P. and in the two others which I have just related, led a physician of this town to conceive, that he should derive benefit from its external application, under a severe head-ache. To this malady he is often incident; and when he has recourse to laudanum for its relief, he generally experiences great subsequent nausea, vertigo, and nervous debility. Being attacked with violent pain above the left eye, where the frontal ramus of the nervus opthalmicus spreads itself, he had the whole forehead repeatedly rubbed with the following ointment:

"R/. Opii. purif. gr. x. camphor. gr. v.

"Ung. Adipis Suillæ, unc. fs. M.

"The camphor dissolves the opium, and renders the composition so smooth, as to fit it for absorption.

"At first the unction, which was applied to a sufficiently large surface, the fore part of the head being bald, seemed to sooth and to assuage the pain. But this comfortable effect soon ceased: the head-ache continued, increased, and terminated only at the usual period.

"The disappointment in this instance ought not, perhaps, to discourage a further trial of the ointment, at the commencement of my friend's next fit of head-ache. But the remedy is more likely to be successful, if the ointment be carefully rubbed on the inside of the legs and thighs, where the lymphatics are far more numerous than on any part of the head. This distribution of the absorbents, may give peculiar efficacy to the anodyne inunction in painful menstruation; to which those females are generally most liable, who suffer extreme faintness, languor, and sickness, from taking opium in the usual forms of its exhibition. And in the mortification of the toes, described by Mr. Pott, the same application is highly worthy of a trial, in conjunction with the internal use of laudanum: for, as opium appears to possess, in some degree, a specific action in this disease, the more fully the system can be imbued with it, without producing stupor, or injuring the energy of the brain, the greater likelihood there must be of arresting the progress of this direful disorder. In a case, about which I was not long since consulted, I observed the gangrene extended itself in streaks up the the inſide of the leg, along the courſe of the abſorbents. If this method of treatment had then occurred to my mind, I ſhould not have heſitated to recommend its adoption."

Being impreſſed with the apparently beneficial effects produced by opium applied externally, in three of the inſtances above related, I felt a ſtrong inclination to adopt the ſame practice, in a caſe of chronic rheumatiſm of ſeven months continuance, which had not yielded to the uſual plans of treatment.

My principal view in this trial was, that it might probably prove a ſubſtitute for the pulv. ipec. comp., which the patient had tried to wean herſelf from, ſeveral times, but in vain, although it had been highly injurious to her general health.

Were this point gained, I was in hopes ſhe would then be able to take a more generous diet, and tonics; without which, her recovery could not be expected.—The following is a brief ſtatement of the principal occurrences.—

May 21, 1799. Mary Caldwell, aged 34, was attacked with lumbago and ſciatica, in November laſt; ſhe was much debilitated and emaciated at that time, by a long continuance of menorrhagia. I was called to viſit her about three months ſince, and found her in a very reduced ſtate, having had very little eaſe or ſleep for ſome time. She obtained temporary relief from pulv. ipec. comp. and topical applications, and has continued to take a doſe of the powder almoſt every night, to the preſent time; not being able to obtain either eaſe or reſt without it. Bark, tincture of guaicum, and calomel in ſmall doſes were of no ſervice, and ſhe continued to decline in her general health, being much harraſſed with pain and flatulence in her bowels, coſtiveneſs, and ſickneſs. Pul. rhei, and kali vitriolatum, were added to the powders, but ſeemed to hinder their anodyne and ſudorific effects. Aperient medicines of different kinds were given; but the relief procured by them was of ſhort duration.

The powders were ordered to be diſcontinued, and ſix grains of opium, mixed with three drachms of lard, to be rubbed on the inſide of one thigh, for thirty or forty minutes at bed time.

May 22. She had not ſo good a night as ſhe had been accuſtomed to have after taking thirteen grains of pul. ipec. comp. at bed time. Twelve grains of opuim, mixed with three drachms of lard were ordered to be rubbed on the inſide of the other thigh at nine o'clock to-night.

May 23. She was reſtleſs the beginning of the night, but ſlept comfortably from one o'clock A. M. till ten. Is better to day than ſhe has been for ſome time time paſt: has not been troubled with ſickneſs and flatulence, and has had two eaſy natural ſtools: her appetite is improved. Pergat.

May 24. Slept but indifferently the beginning of the laſt night; but pretty well afterwards: appetite continues better: has had two ſtools. Fifteen grains of opium mixed with three drachms of lard, were ordered to be rubbed in to-night an hour ſooner than uſual.

May 25. She fell aſleep ſoon after the ointment had been applied, and had a good night. The ſame plan was ordered to be continued.

May 28. The pains are not ſo ſevere, nor do they continue quite ſo long when they come on; but ſhe ſeldom ſleeps till towards one o'clock.. I deſired to ſee the ointment, and found the opium which had been uſed on the 25th and ſince that time, was not powdered ſufficiently fine.

The pained parts were ordered to be rubbed morning and evening, with ſome of the following liniment, and I deſired the friction might be continued fifteen or twenty minutes at a time, that a conſiderable portion of the liniment might be abſorded. R/. Lin. Sapon. Æther. Tinct. Opii *aa*. unc. i. M. R/. Infus. Cort. Peruv. Vin. Rub. *aa*. unc. vi. Tinct. Lavend. Comp. unc. i. M.—Cap. cochl. iij larg. ter die.

June 1. She has walked out twice, and is not worſe.

The ointment, each portion containing fifteen grains of opium in fine powder, was ordered to be continued.

From the facts above recited, I think I am warranted in drawing the following inferences; 1ſt. That opium, when diligently applied externally, ſo as to be abſorbed by the lymphatics, has powerful effects in allaying irritation, removing ſpaſm, and procuring ſleep. 2dly. That it is capable of producing theſe happy effects, where the exhibition of it internally had not the ſame ſalutary operation. 3dly. That this mode of introducing it into the ſyſtem, may be reſorted to with advantage, when it cannot be given internally, or when it will not ſtay on the ſtomach.

Viewing the ſubject in this light, a wide field ſeems to lie open for inveſtigation, which, if cultivated, may poſſibly lead to important improvements in the practice of medicine.

As opium, applied externally, poſſeſſes the peculiar advantages above mentioned; would it not be worth while to try its effects, in this way, in hydrophobia and in tetanus?—Unfortunately the former is juſtly ranked among mortal diſeaſes, and the latter generally proves fatal. Every other mode of treatment having been unſucceſsful, is a ſufficient reaſon for adopting ing fome new plan of treatment, provided it have reafon and probability to recommend it: and opium, on account of its foothing antifpafmodic properties, feems ftrongly and peculiarly indicated in thefe difeafes.

But it may probably be urged, that the modus operandi muft be the fame, whether opium be given internally, or applied externally by abforption; and, therefore, as it has not been efficacious when taken by the mouth, we cannot expect it to be fo when applied externally: but experience, the only true teft by which to try every hypothefis, feems at variance with this: at leaft, the facts contained in the cafes above recited, militate as far as their authority extends, againft the idea of the modus operandi being the fame, the effects produced being fo different.

Another ftrong argument in favour of the external application of opium in hydrophobia, is, that fhould it be found to be poffeffed of properties capable of counteracting the effects of the virus, it will be of peculiar advantage, that we can introduce it into the habit by the fame fyftem of veffels which convey the virus into the circulation: and it is worthy of confideration, that fhould my hopes and expectations of fuccefs be difappointed, it does not feem probable that any mifchief can be done by this practice, efpecially if I am right in fuppofing the difeafe to belong to the afthenic clafs.

Whether opium applied externally, may or may not prove an antidote to the canine virus, it would, I think, be unpardonable to rely exclufively on thofe plans of treatment, which have been fo often tried, and always without fuccefs.

Whoever may think proper to make trial of this mode of treatment, fhould fee that the powder, if that be preferred to the tincture,* is made as fine as poffibe, and that it is rubbed in, in large quantities,† (the difeafe being fo violent and fo rapid in its progrefs) on the infide of the legs and thighs, and alfo on the part where the bite was inflicted in hydrophobia, or a wound in tetanus; repeating it at fhort intervals,‡ till it produces fome fenfible effect on the fyftem in general.

That the other parts of the treatment may coincide, as far as poffible, with the foothing plan here propofed, it will be neceffary to keep patients labouring under

* Would it not be adviseable, till we know which of these preparations is absorbed with the greatest facility, to use both either alternately, or together?

† Not less than a drachm of the powder, or an ounce and a half of the tincture, to an adult subject.

‡ I should imagine an hour would be a sufficient length of time, to allow to intervene between the frictions.

under hydrophobia or tetanus as quiet as poſſible, and to avoid every thing which can tend to agitate and alarm, excite uneaſy ſenſations, or bring on a return of the ſpaſms. In conformity to this intention, inſtead of importuning the unfortunate ſufferer to ſwallow medicines, or liquids, of which he has ſo great a dread, glyſters might be given every three or four hours, perhaps with advantage, to ſupport the ſtrength, conſiſting of good broth, or milk, with thirty or forty drops of laudanum in each. Should the patient aſk for food or drink, warm milk or jelly, or mulled wine, may be given from a tea-pot ſpout; but on no account ought liquids to be brought into his ſight, or to be diſturbed or agitated within his hearing. For ſimilar reaſons, I ſhould think it improper to aſk the hydrophobic patient any queſtions reſpecting the dog or the bite. He ſhould be carefully guarded againſt the effects of cold (ſome patients having expreſſed almoſt as great an averſion to a ſtream of cold air, as to liquids), by wearing a flannel ſhirt next to his ſkin, and by wrapping his legs and feet in warm flannel, or putting him on woollen ſtockings, and by warming his apartment moderately with a fire or a ſtove. If it be neceſſary to remove him to a hoſpital, or elſewhere, after the coming on of the diſeaſe, he ſhould be warmly clothed, and removed as eaſily and carefully as poſſible.

Should experience ſhew, that the plan here propoſed is capable of curing, or even of mitigating the ſufferings of this unfortunate claſs of patients, a moſt valuable acceſſion to medical knowledge will be gained; but we ought not to be ſatisfied with a few trials of this practice, I think, ſhould the reſult be unfavourable.

